# Oiling of American white pelicans, common loons, and northern gannets in the winter following the Deepwater Horizon (MC252) oil spill

**DOI:** 10.1007/s10661-019-7925-y

**Published:** 2020-03-17

**Authors:** J. D. Paruk, I. J. Stenhouse, B. J. Sigel, E. M. Adams, W. A. Montevecchi, D. C. Evers, A. T. Gilbert, M. Duron, D. Long, J. Hemming, P. Tuttle

**Affiliations:** 1 St. Joseph’s College, 278 Whites Bridge Road, Standish, ME 04084 USA; 2 Biodiversity Research Institute, 276 Canco Road, Portland, ME 04103 USA; 3 Nevada State College, 1300 Nevada State Dr, Henderson, NV 89002 USA; 4 Memorial University of Newfoundland, 230 Elizabeth Ave., St. John’s, NL A1C 5C7 Canada; 5 U.S. Fish and Wildlife Service, 1208 Main St. B, Daphne, AL 36526 USA

**Keywords:** American white pelican, Common loon, Deepwater horizon oil spill, Northern gannet, Oiling

## Abstract

The Natural Resource Damage Assessment and Restoration Trustees for the Deepwater Horizon oil spill assessed the external oiling of migratory bird species dependent on open water in the Gulf of Mexico following the aforementioned spill. The assessment was designed to evaluate birds that use open water during the winter within 40 km of the Gulf shoreline. We focused on the American white pelican (*Pelecanus erythrorhynchos*), common loon (*Gavia immer*), and northern gannet (*Morus bassanus*). Point counts (pelican, loon) or strip transects (gannet) were used and each target species was assessed for oiling (unoiled, trace, light, moderate, or heavy amounts) and photographed. Due to distance at sighting and/or poor visibility, not all visible birds were assessed. The percentage of birds oiled varied by species, with the common loon being the highest (23.6%), followed by American white pelican (16.9%), and northern gannet (6.9%). Most of the American white pelicans and common loons had trace (83% and 72%, respectively) or light levels (11% and 24%, respectively) of oiling. The northern gannet had just trace levels of oiling. Some pelicans (6%) and loons (4%) had moderate amounts of oiling. Based on expert derived-mortality estimates and our estimates of oil exposure, we used Monte Carlo simulations to predict expected decreases of 2.5%, 4%, and 11% in the observed population for the northern gannet, American white pelican, and common loon, respectively. While these values are underestimates of the true values given the long time lag (10–12 months) between the oil spill and the assessment, these data represent some of the few estimates of exposure for these species and describe minimum risk estimates to these species.

## Introduction

Seabirds are excellent indicators of oil pollution because they spend most of their time in marine environments on the ocean surface where oil tends to disperse and persist after spills (Furness and Camphuysen 1997; Montevecchi 1993, 2001). Consequently, they are at greater risk of oil exposure than other birds. Oil exposure interferes with the zipping mechanism of feathers critical for waterproofing and insulation, allowing water to penetrate to the skin resulting in heat loss, which can lead to hypothermia and death (Newman et al. 2000; Albers 2006; O’Hara and Morandin 2010). Birds can ingest oil directly from the water, from their food, and from preening contaminated feathers (Burger 1997). Some estimates suggest that birds ingest 50% of the oil on their feathers within 8 h of exposure (Hartung and Hunt 1966). Ingestion of oil exposes birds to polycyclic aromatic hydrocarbons (PAHs), highly toxic components of crude oil and known carcinogens (Albers 2006). In addition, PAH exposure is linked to numerous pathological effects, such as anemia, gut damage, renal and liver damage, alterations in immune and endocrine function, and weight loss (Peakall et al. 1981; Fry and Lowenstine 1985; Leighton 1993; Jenssen 1994; Yamato et al. 1996; Briggs et al. 1997; Burger and Tsipoura 1998; Trust et al. 2000; Paruk et al. 2016; Fallon et al. 2017; Harr et al. 2019). Exposure to sublethal oiling levels puts more birds at risk, potentially leading to lower fitness (Golet et al. 2002; Alonso-Alvarez et al. 2007).

As part of the avian injury assessment for the Deepwater Horizon (DWH) oil blowout, the US Fish and Wildlife Service, together with the Trustees, assessed the oiling of migrant bird species on the open water in the first winter (2011) following the spill. The DWH spill occurred roughly 80 km off the Louisiana shore and the surface oil covered > 175,000 km^2^ in the Gulf of Mexico (Beyer et al. 2016). The assessment was designed to evaluate birds that spend the winter months from nearshore to offshore (up to 40 km) of the Gulf of Mexico shoreline. We focused on three target species that fit those criteria and that were relatively common and widespread: the American white pelican (*Pelecanus erythrorhynchos*), which tends to inhabit coastal waters (King and Michot 2002); the common loon (*Gavia immer*), which tends to inhabit nearshore waters (Jodice 1993; Kenow et al. 2009); and the northern gannet (*Morus bassanus*), which inhabits both nearshore and offshore waters. These target species all have deferred maturity, high adult survival rates, and low reproductive rates, so any impacts that lower adult survival could have long-lasting population level effects (Furness and Monaghan 1987). The objective of this study was to estimate the proportion of birds oiled to various degrees at representative areas throughout the study area. We used expert opinion (IEc 2015) to estimate (1) the expected mortality rates for each species due to the level of oiling observed, and (2) in combination with the oil exposure data, the relative influence of oil exposure on target species populations.

## Methods

### Sampling design

**Fig. 1:**
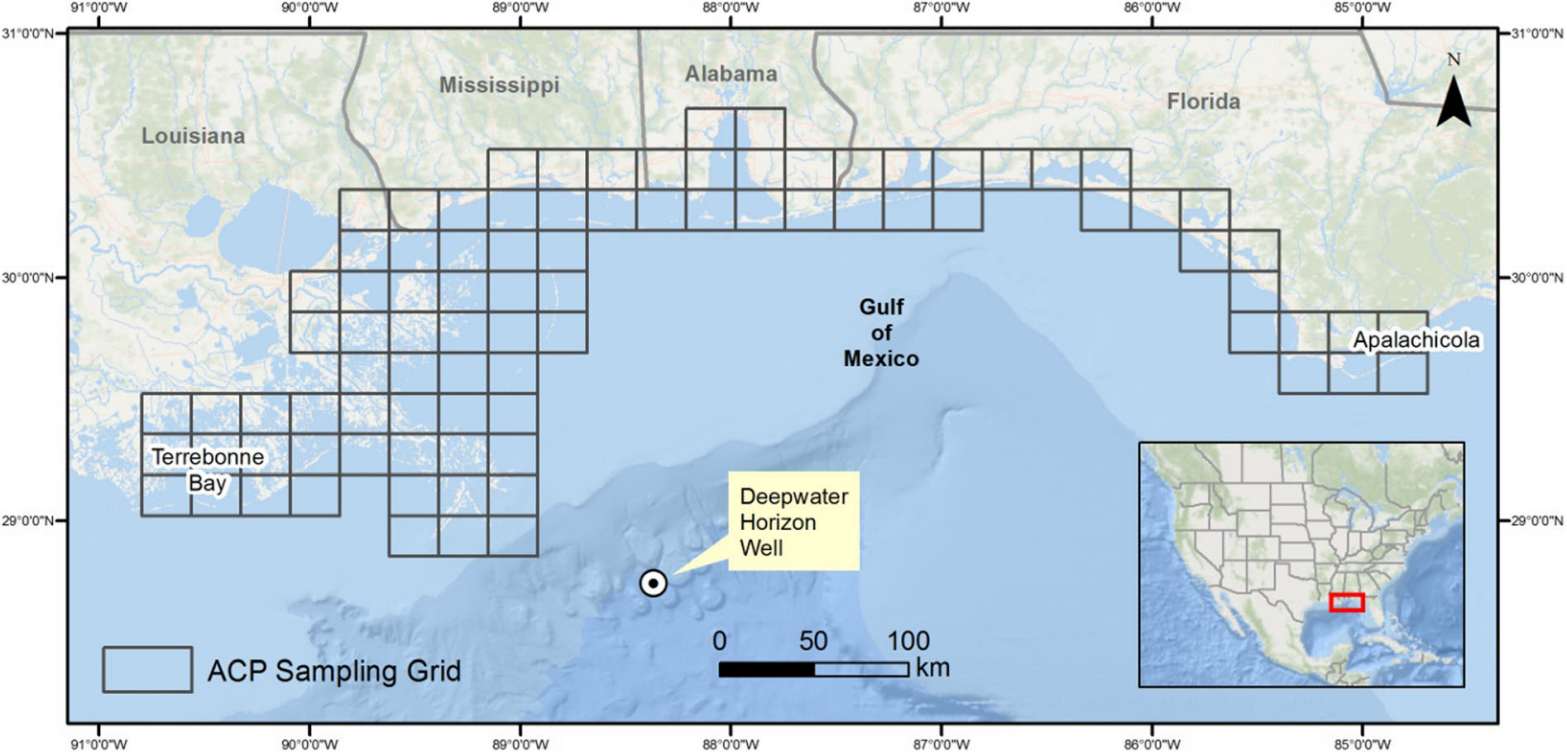
Study area and US Coast Guard Area Contingency Plan grids where boat- and land-based surveys for external oiling of American white pelicans, common loons, and northern gannets were conducted, January–March 2011

The study area ranged from Terrebonne Bay, Louisiana (LA), to Apalachicola, Florida (FL; Fig. 1), as this area in the northern Gulf of Mexico was most influenced by the Deepwater Horizon oil spill (Michel et al. 2013). Different sampling designs were used for the nearshore species (pelicans, loons), and for gannets that range further offshore. The designs were developed to sample the study area, detect as many individuals of the species of interest as possible, and acquire a reliable estimate of the degree of external oiling if any for each individual (Briggs et al. 1985; Henkel et al. 2007).

### Pelicans and loons

The entire study area, from coastline to 5 km out at sea was organized by Area Contingency Plan (ACP) grid cells as adopted by the LA Coast Guard (Fig. 1). The pelican team attempted to survey 25% of all ACP grid cells from Terrebonne Bay, LA, to Mobile Bay, Alabama (AL), every 5 days, given suitable viewing conditions. The loon teams surveyed at least 20% of the ACP grid cells from Terrebonne Bay to Mobile Bay, and at least 10% of the ACP grid cells in the area between Mobile Bay and Apalachicola, Florida (FL), every 5 days. Priority was given to completing surveys first in areas with target species concentrations that were documented to be moderately or heavily oiled (local experts, eBird).

Pelican and loon surveys were conducted primarily using boat-based observations (21 ft bay boat) within nearshore habitats, such as bays and coves, as well as along barrier islands, primarily in LA and Mississippi (MS); a few surveys were also conducted from shore. Once biologists entered a specific ACP grid, they immediately looked for target species and if any were observed, approached them (to within 30–70 m) to visually assess each individual for oiling. To assess oiling rates, observers used either 8 × 40 or 10 × 50 binoculars from boats or spotting scopes (20–60×) from land. Every attempt was made to photo document all birds assessed and the level of oiling. Pelican and loon surveys were conducted from 17 February to 15 April 2011 and 17 February to 15 March 2011, respectively.

### Gannets

**Fig. 2:**
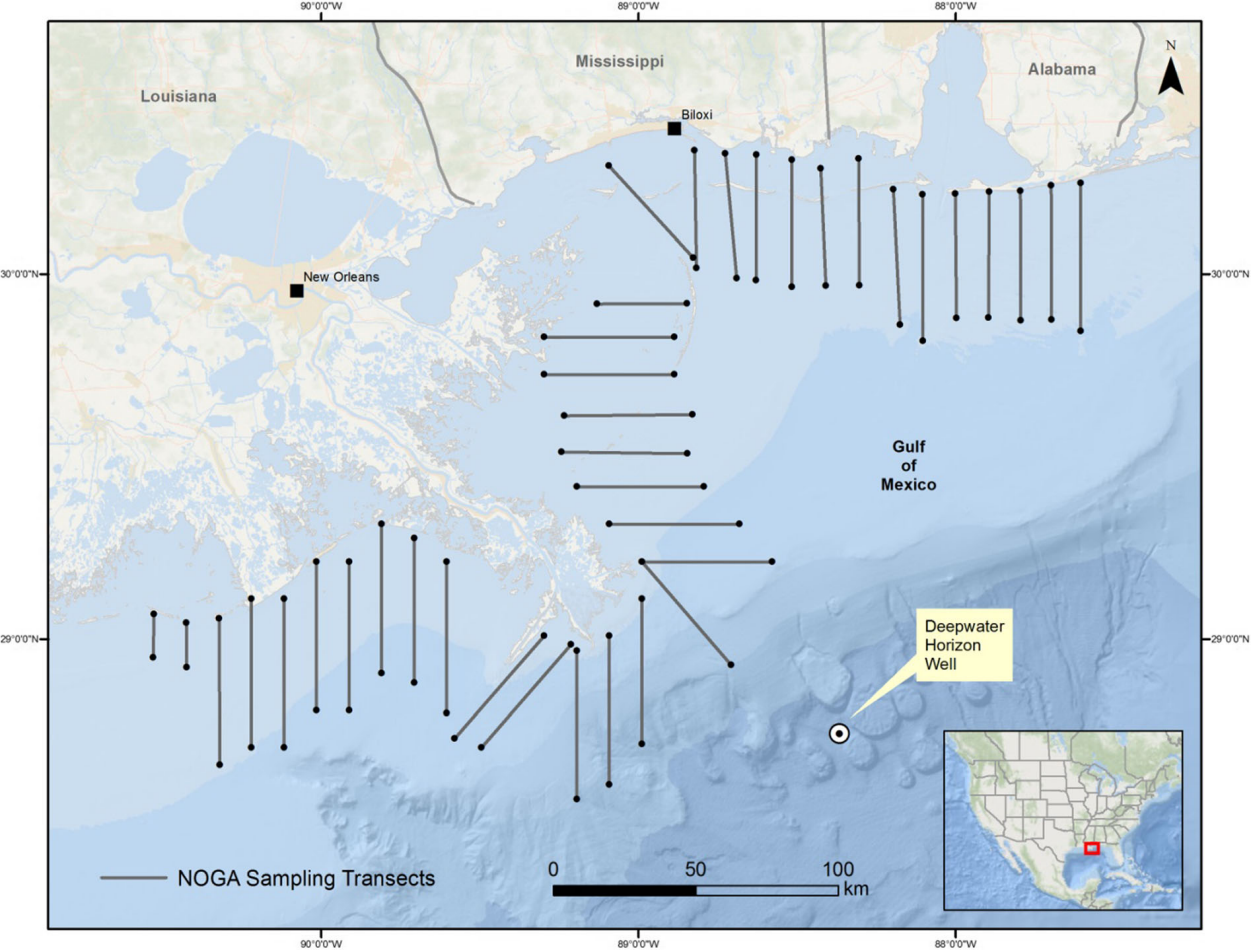
Survey transect design for northern gannets in the northern Gulf of Mexico, 17 February 2011–16 March 2011

Northern Gannets were assessed using systematic boat-based strip transects. Transects were spaced every 10 to 20 km, beginning from shore and extending out to 40-km offshore. They ran either perpendicular to shore, parallel to a selected set of grid lines, or used a hybrid approach (Fig. 2). Nearshore to offshore, boat-based surveys spanning the western half of the study area were conducted during the peak of northern gannet occupancy in the Gulf of Mexico (17 February to 16 March 2011).

When aggregations of foraging or loafing flocks of northern gannets were observed (> 20 individuals), observers determined, based on the gannet’s general behavior, whether the aggregation was expected to remain in place long enough to allow for assessment. If so, strip transects were paused so the vessel could move off transect and approach the aggregations and conduct a point count. Once that point count was completed, the vessel returned to transect and continued the strip-transect methods. Observations were conducted from an elevated viewing platform that provided an unobstructed view of the water. Gannets and other seabirds within a 90°, bow-beam arc and within 300 m of one side of the ship were recorded, following standard at-sea survey protocols (Tasker et al. 1984), and including recording the distance of the seabirds from the observer. Observations were recorded using dLOG3, real-time data entry and mapping software for biological surveys (Ford 2009). Individuals observed within 100 m of the ship were given the highest priority for assessment of oil exposure. Every attempt was made to photo document all birds assessed and the level of oiling.

### Oiling

We described oil exposure over space and time in the northern Gulf by identifying where ACP grid cells contained at least one visibly oiled bird from each target species. Oil exposure was summarized for all three target species by quantifying the percentage of each surveyed population assigned to each exposure category.

During surveys, bird oiling levels were categorized as “None,” “Trace” (greater than 0, but < 5% of the bird’s body surface), “Light” (6–20%), “Moderate” (21–40%), and “Heavy” (> 40%). Oiling rates were only estimated for birds when more than 50% of the body surface could be clearly observed. Each bird that was clearly observed was assigned an oil exposure category based on visual and photographic inspection. A laser range finder was used to measure the distance between the observer and birds observed; however, measurements were not made for each individual bird if found in a group. Observations were geo-referenced using GPS coordinates.

### Mortality estimates

The Trustees organized an expert panel of scientists with bird experience related to oil spills and/or species of the northern Gulf of Mexico to consider the influence of external oiling on the mortality and reproduction of various birds (IEc 2015). This information was used by an avian injury expert in conjunction with avian toxicity testing data specific to the Deepwater Horizon oil spill to estimate the fate of birds that were observed alive after the spill, but with external oiling (IEc 2015, Ziccardi 2015). In the present assessment, we used the same categories of visible oil that the panel considered, which were based on a similar assessment for the M/T Athos I oil spill (Athos 2007). In addition, the panel was charged with considering the effects of season on the fate of oiled birds. The mortality estimates as determined by the panel for each target species with trace, light or moderate oiling, respectively, were as follows: American white pelican = 0–30%, 15–70%, and 40–100%; common loon = 0–60%, 80–90%, and 90–100%; and northern gannet = 0–40%, 30–80%, and 70–100%. Note our mortality estimates are not of total population decline, we lack the information for that, but rather an estimate of the proportion of birds surveyed that died due to oil exposure.

### Monte Carlo simulation

To provide context for the expert-derived estimates of mortality, we used a Monte Carlo simulation (MCS) to determine how much of a decrease in target species populations would have been expected based on the estimates of oil exposure. For each iteration of the simulation, we began with the total number of detections of each target species then assigned a subset of the total to oil exposure categories at the exposure rates documented in this study using a random binomial draw. For each species, we then estimated the probability of mortality at each exposure level using the Trustee expert panel data. Mortality estimates were provided as ranges rather than estimates of central tendency with a variance estimate, so we used a uniform distribution to randomly select any value within the range for each simulation. Last, we used another binomial draw to determine the number of birds that survived their oil exposure in each exposure category based on the total number of birds in that exposure category and the randomly selected mortality probability. We ran these simulations for 100,000 iterations (the point at which the results appeared to be properly stable across multiple seeded attempts) for each species using the R statistical computing environment (R Core Team 2015; v 3.2.2); results were summarized as the median and 95% confidence interval of the estimated population decrease due to oil exposure.

## Results

### American white pelican

A total of 483 surveys were conducted, 396 (82.0%) in LA, 10 (2.1%) in MS, and 77 (15.9%) in AL. During the surveys, 2407 American white pelicans were observed and most met the criteria for oil exposure assessment (85.2%, *n* = 2050; Table 1). Oil was observed on 16.9% (347/2050) of the assessed pelicans with most showing trace (83%*,* 289/347) or light (11%, 38/347) extents, and some with moderate oiling levels (6%*,* 20/347; Table 1). Overall, the highest percentage of oiled birds by state in descending order was AL (34%), LA (15%), and MS (7%); ACP grid cells with at least one bird having oil were common and distributed across the study area. Given the level of oiling and the corresponding mortality estimates, the MCS predicted a population decrease of 3.7% (95% CI 1.6–5.8%).

**Table 1:** Total number of each species observed, number and percentage of total assessed for oiling, the number and percentage (of assessed) visibly oiled, and a breakdown of the extent of oiling, including the number and percentage (of assessed) in each oiling category

Species	State	No. observed	No. assessed	No. visibly oiled	Trace	Light	Moderate
American white pelican	Louisiana		1819	276 (15%)	231 (84%)	25 (9%)	20 (7%)
	Mississippi		27	2 (7%)	2 (100%)	0	0
	Alabama		204	69 (34%)	56 (81%)	13 (19%)	0
	Total	2407	2050 (85%)	347 (17%)	289 (83%)	38 (11%)	20 (6%)
Common loon	Louisiana		64	21 (33%)	14 (67%)	6 (28%)	1 (5%)
	Mississippi		13	1 (8%)	1 (100%)	0	0
	Alabama		9	1 (11%)	1 (100%)	0	0
	Florida		20	2 (10%)	2 (100%)	0	0
	Total	1148	106 (9%)	25 (23%)	18 (72%)	6 (24%)	1 (4%)
Northern gannet		2436	87 (4%)	6 (7%)	.	.	.
All species	Total	5991	2243 (37%)	378 (17%)	307 (81%)	44 (12%)	21 (5%)

### Common loon

Forty-five loon surveys were carried out, 20 (44.4%) in LA, 11 (24.4%) in MS, seven (15.6%) in Al, and seven (15.6%) in FL (Table 1). At least one common loon was observed in over half of the ACP grid cells surveyed (*n* = 24/45, 53.3%). Visibly oiled loons were observed in 11 ACP grid cells (24.4%, 11/45). The distribution of oiled common loons, however, was not uniform across ACP grid cells or states, with most oiled birds being recorded in LA.

Field observers tallied 1148 common loons. Following the study protocol, however, only loons that were clearly visible (> 50% of body surface) were assessed. Thus, a total of 106 (9.2%) common loons were assessed for oiling. Most did not show visible oiling (76.4%; *n* = 81), while 25 (23.6%) showed visible oiling. Of these, 18 had trace levels (72.0%), six had light levels (24.0%), and one showed moderate oiling (4.0%; Table 1). Overall, loons observed in LA had the highest oiling rate (33.3%; *n* = 64), followed by AL (11.1%; *n* = 9), FL (10.0%; *n* = 20), and MS (7.7%; *n* = 13). Given the level of oiling and the corresponding mortality estimates, the MCS predicted a population decrease of 10.7% (95% CI 6.0–15.8%).

### Northern gannet

A total of 36 survey transects were completed, and ~ 30% of two additional transects were completed. In total, more than 1400 transect km were surveyed (Fig. 2). Point counts (*n* = 26) were conducted opportunistically when aggregations of northern gannets and other birds occurred within sight of observers from their transect position.

A total of 2436 northern gannets were recorded during offshore surveys, with only 87 (3.6%) of them observed clearly enough to be visually assessed for oiling (Table 1). Of the assessed birds, the great majority (95.4%; *n* = 83) were not visibly oiled and a few (4.6%, *n* = 4) showed trace levels of oiling. After reviewing the photographs, two additional birds were determined to be visibly oiled, making the oiling rate of northern gannets 6.9% (Table 1). Given the level of oiling and the corresponding mortality estimates, the MCS predicted a population decrease of 1.4% (95% CI 0.1–2.8%) if all NOGAs were of trace exposure, and 3.4% (95% CI 0.3–6.7%) if they could be in any exposure category.

## Discussion

### Oiling rates

Observations of oiling rates for American white pelicans, common loons, and northern gannets were made in February–March 2011, almost a year after the Deepwater Horizon oil spill originated, and 7 months after the well was capped and new oil stopped entering the system. By this time, much of the surface oil from the spill was removed by a variety of remediation responses (e.g., dispersants, skimming, containment booms), had evaporated, reached land, or settled on the sea floor sediment (Atlas and Hazen 2011; Allan et al. 2012). Despite much of the oil being inaccessible to seabirds, we still observed oiled birds across the study area (LA, MS, AL, FL; Table 1), indicating widespread impact of the oil on waterbirds. During April 2011, a number of northern gannets that appeared to have oiled plumage were observed in the colony at Cape St. Mary’s, Newfoundland, Canada (WAM pers. obs.).

Several factors may have affected our estimation of oiling exposure. The level of oiling in the three target species likely reflects their foraging behavior and other species-specific factors that influenced our ability to detect oil on birds. For example, species that do not forage in the sediment would be less likely to contact oil that had settled there. Our estimates do not incorporate how often oil was not detected on an individual when it was present, leading to an underestimation of oil exposure estimates and mortality rates. A further complicating factor in determining oiling rates is the proportion of white plumage typically found in the target species. Oiled non-white plumage can be much more difficult to see, particularly from a distance, and each of the target species had variable proportions of white plumage during the study period. Finally, birds exposed to oil have a higher chance of mortality than unexposed birds, thus removing them from the sampled population. Given these sampling biases, the oiling rate data presented here should be considered underestimates of the true rate.

American white pelicans were relatively easy to assess for oil given their white plumage and tendency to rest on land (85% assessment rate). These factors, in addition to their preference for foraging in the shallows where tar balls and other oil could accumulate (Tao et al. 2011), likely explain the relatively higher detection of oiled individuals sampled (17%; Table 1). Unlike American white pelicans, northern gannets are plunge divers, often foraging far from shore, and are not in contact with sea floor sediments, which may explain why they were the species with the lowest rate of oiling (6.9%). It is also possible the mottled plumage of immature northern gannets masked the presence of oil on some birds. In addition, gannets were observed entirely at sea and most often at considerable distance where it is difficult to clearly see over 50% of their body, resulting in only 4% of individuals being assessed for oil. Like gannets, a low percentage of loons were assessed for oil (9%) for similar reasons (i.e., immatures have significant non-white plumage, and the difficulty observing > 50% of their body at any one time). Despite these factors, common loons had the highest level of oiling (23.6%) among the three focal species. We suspect the high oiling rate in loons compared with gannets is because loons will switch to bottom-dwelling prey (e.g., invertebrates, such as crab and shrimp) instead of fish when water is turbid (Daub 1989; Barr 1996, JP pers. obs.), conditions that existed in the Gulf of Mexico that winter (0.5-m secchi disc, Paruk et al. 2014). Thus, loons likely came in contact with oil when searching for prey which is consistent with the observation that most of the observed oil on loons was on their feet or belly (DL, pers. obs.).

### Potential fate of oiled birds

Although most of the target waterbirds we assessed were unoiled (AMPR 83%, COLO 77%, and NOGA 93%), these surveys occurred 9 months post-blowout and individuals that were previously in the moderate to heavy oiling category may have perished, representing acute mortality, and were removed from the population prior to surveying. Even birds with trace to light oiling levels can experience mortality (Ziccardi 2015). The cumulative effects of oiling can eventually exhaust body energy stores to a point that even trace to lightly oiled birds cannot maintain physiological function and homothermia (Ziccardi 2015). Oiling reduces insulation allowing water to reach the bird’s thin skin, increasing vulnerability to hypothermia. To offset this thermal loss, birds normally increase their basal metabolic rate, which can deplete stored body fat and possibly starvation. Ingestion of oil can lead to anemia (Leighton 1993; Yamato et al. 1996, Fallon et al. 2017) which can affect aerobic performance and since all these three species are migratory, any reduction in oxygen carrying capacity during flight is likely to have adverse effects. In addition, because loons and gannets are divers, reduction in oxygen carrying capacity could compromise foraging abilities.

Oiling is just one of many stressors these target species experience on their wintering grounds. Cleanup activities following the spill were ongoing during the sampling period and the additional human disturbance was found to have impacted the fattening rate of shorebirds (Henkel et al. 2014), and likely also affected our target species. Ultimately, all of the target species undergo molt, have to find food, avoid predators, endure storm events, etc., and additional environmental stressors act synergistically (Whitehead 2013) exacerbating adverse effects.

Based on the recommendations of an expert panel (the best available data on oil-related mortality rates for these species), our simulation work provided some context for the population implications of the exposure rates documented in this study. Of the three target species, based on the mortality estimates decided on by the expert panel for each species (Ziccardi 2015) and the observed oiling rates, the common loon had the highest estimated population decline (11%), followed by the American white pelican (4%), and the northern gannet (1.4–3.4%). Common loons combined high oiling rates with the highest concern for oil-related mortality by the expert panel, while less vulnerability or less observed oil exposure resulted in lower estimated declines in the other species. To convert these data into estimates of total population decline, one would have to estimate oil exposure rates to species throughout the blowout (not just 7–11 months after) then conduct a field study to estimate the effects of oil exposure on seabird mortality. Despite such shortcomings, our analysis is useful for estimating the effects of oil exposure onto our observed populations based on the best available expert opinion.

## Conclusions

Thousands of American white pelicans, common loons, and northern gannets were observed in the northern Gulf of Mexico during the winter following the Deepwater Horizon spill. Despite being 7–11 months after the origin of the blowout, the oiling rates of these waterbirds ranged from at least 7 to 24% and these oiling rates likely caused significant mortality in these populations (1–11% above unoiled populations). Categories of oiling were most often trace, though both light and moderate oiling were also observed. Any American white pelicans, common loons, and northern gannets that were moderately (90–100% mortality) to heavily oiled (95–100% mortality) had probably died by the time the surveys were being conducted and may explain why so few birds were observed in these oiling level categories. Birds with trace, light, or moderate amounts of oiling both inhaled and ingested the oil, and chronic exposure to petroleum can have numerous sublethal effects, which, depending on the concentrations of the oil and duration of exposure, may ultimately have population-level effects (Albers 2006; Esler et al. 2010). The potential influence of behavior (congregating far offshore, gannets) or plumage coloration (predominately dark, juvenile gannets) on determining oiling rates warrants additional consideration.
